# Efficient Isolation of Outer Membrane Vesicles (OMVs) Secreted by Gram-Negative Bacteria via a Novel Gradient Filtration Method

**DOI:** 10.3390/membranes14060135

**Published:** 2024-06-06

**Authors:** Ning Li, Minghui Wu, Lu Wang, Mengyu Tang, Hongbo Xin, Keyu Deng

**Affiliations:** The National Engineering Research Center for Bioengineering Drugs and the Technologies, Institute of Translational Medicine, Jiangxi Medical College, Nanchang University, Nanchang 330031, China; 15179421660@163.com (M.W.); 407400210001@email.ncu.edu.cn (L.W.); 405600210073@email.ncu.edu.cn (M.T.); xinhb@ncu.edu.cn (H.X.)

**Keywords:** outer membrane vesicles, bacterial extracellular vesicle, gradient filtration method, *Escherichia coli* Nissle 1917, macrophages

## Abstract

Bacterial extracellular vesicles (bEVs) secreted by Gram-negative bacteria are referred to as outer membrane vesicles (OMVs) because they originate in the outer membrane. OMVs are membrane-coated vesicles 20–250 nm in size. They contain lipopolysaccharide (LPS), peptidoglycan, proteins, lipids, nucleic acids, and other substances derived from their parent bacteria and participate in the transmission of information to host cells. OMVs have broad prospects in terms of potential application in the fields of adjuvants, vaccines, and drug delivery vehicles. Currently, there remains a lack of efficient and convenient methods to isolate OMVs, which greatly limits OMV-related research. In this study, we developed a fast, convenient, and low-cost gradient filtration method to separate OMVs that can achieve industrial-scale production while maintaining the biological activity of the isolated OMVs. We compared the gradient filtration method with traditional ultracentrifugation to isolate OMVs from probiotic *Escherichia coli* Nissle 1917 (EcN) bacteria. Then, we used RAW264.7 macrophages as an in vitro model to study the influence on the immune function of EcN-derived OMVs obtained through the gradient filtration method. Our results indicated that EcN-derived OMVs were efficiently isolated using our gradient filtration method. The level of OMV enrichment obtained via our gradient filtration method was about twice as efficient as that achieved through traditional ultracentrifugation. The EcN-derived OMVs enriched through the gradient filtration method were successfully taken up by RAW264.7 macrophages and induced them to secrete pro-inflammatory cytokines such as tumor necrosis factor α (TNF-α) and interleukins (ILs) 6 and 1β, as well as anti-inflammatory cytokine IL-10. Furthermore, EcN-derived OMVs induced more anti-inflammatory response (i.e., IL-10) than pro-inflammatory response (i.e., TNF-α, IL-6, and IL-1β). These results were consistent with those reported in the literature. The related literature reported that EcN-derived OMVs obtained through ultracentrifugation could induce stronger anti-inflammatory responses than pro-inflammatory responses in RAW264.7 macrophages. Our simple and novel separation method may therefore have promising prospects in terms of applications involving the study of OMVs.

## 1. Introduction

Gram-negative bacteria can release bacterial extracellular vesicles (bEVs). bEVs secreted by Gram-negative bacteria are referred to as outer membrane vesicles (OMVs) because they originate in the outer membrane [1,2]. OMVs are spherical vesicles of lipid membrane structures [3]. OMVs cannot replicate, and they range in size between 20 and 250 nm [1,3]. Research has shown that the formation and release of OMVs is a delicate and orderly regulatory process in bacteria. OMVs contain lipopolysaccharide (LPS), peptidoglycan, proteins, lipids, nucleic acids, and other substances derived from their parent bacterial cells and participate in the transmission of information to host cells [4,5,6]. OMVs mediate the interaction between bacteria and host cells, submit bacterial antigens, and regulate the immune function of host cells [7,8,9]. OMVs have been shown to regulate certain functions of macrophages. Owing to the differences in the bacterial origins and the diversities in the contents of OMVs, they have pro-inflammatory and anti-inflammatory effects on macrophages. For example, a study indicated that OMVs derived from *Neisseria meningitidis* can induce adaptive immune responses by activating macrophages [10]. Another related study reported that *Neisseria meningitidis*-derived OMVs could stimulate macrophages to produce pro-inflammatory cytokines such as interleukins (ILs) 1β and 6, as well as tumor necrosis factor α (TNF-α) [11]. Wang et al. reported that *Helicobacter pylori*-derived OMVs could promote the production of IL-6 in human peripheral blood mononuclear cells [12]. Liu et al. reported that *Salmonella*-derived OMVs could induce the production of TNF-α and nitric oxide (NO) in mouse macrophages [13]. Zhang et al. reported that *Haemophilus parasuis*-derived OMVs could significantly increase the IL-1β mRNA expression and secretion in mouse mononuclear macrophage cells (J774A.1) [14]. In addition to pro-inflammatory effects, OMVs can also have anti-inflammatory effects. For instance, *Helicobacter pylori*-derived OMVs induced human peripheral blood mononuclear cells to produce the anti-inflammatory cytokine IL-10, which limited inflammation and promoted bacterial survival [15]. Sun et al. reported that *Pasteurella multocida*-derived OMVs could trigger the secretion of pro-inflammatory cytokines (IL-1β, IL-6, and TNF-α), and anti-inflammatory cytokines (IL-10 and TGF-β1) in RAW264.7 cells [16].

OMVs show promising prospects in terms of applications in the biomedical field. The use of OMVs mainly reflects the following three aspects. First, OMVs can induce robust changes in terms of cellular immunity and humoral immune response capability, so they may have applications as adjuvants in vaccines [17]. Second, OMVs do not replicate, and they express a variety of virulence factors, so they can be used as vaccine antigen candidates during vaccine development [18,19,20]. Third, they have unique nanoscale structures, strong loading capacities, and good biocompatibility and can be used as drug carriers for disease treatment [21,22]. Compared with extracellular vesicles derived from animal cells, which suffer from drawbacks such as low yield and difficulty regarding industrial-scale production, OMVs may be scalable to mass-production levels. This is mainly because bacteria have the ability to reproduce rapidly and can be cultured at high densities. Currently, ultracentrifugation and density gradient centrifugation represent the most commonly used techniques to separate and purify OMVs [23]. However, these methods are labor-intensive and time-consuming and require the purchase of expensive ultracentrifuge instruments. At present, there remains a lack of fast, efficient, and convenient methods to separate OMVs, which greatly limits research on the subject.

In this study, we developed a novel gradient filtration method to separate OMVs. Using a combination of filter membranes with different pore sizes (300 nm and 100 kDa), we were able to create a system wherein small molecular substances passed through while larger particles were retained, thereby achieving the effect of separation. This method has the advantages of being fast, convenient, and low-cost and achieving industrial-scale production of OMVs while maintaining their biological activity. We compared the gradient filtration method with traditional ultracentrifugation for the isolation of OMVs from probiotic *Escherichia coli* Nissle 1917 (EcN) bacteria. Then, we used RAW264.7 macrophages as an in vitro model to study the influence on the immune function of EcN-derived OMVs obtained through the gradient filtration method. We discovered that the EcN-derived OMVs were efficiently obtained via our gradient filtration method. Furthermore, the production of OMVs achieved with the gradient filtration method was about twice as efficient as that obtained via traditional ultracentrifugation. We found that the EcN-derived OMVs obtained through the gradient filtration method were successfully taken up by the RAW264.7 macrophages and induced them to secrete pro-inflammatory cytokines such as TNF-α, IL -6, and IL-1β, as well as the anti-inflammatory cytokine IL-10. Interestingly, we also found that the EcN-derived OMVs predominantly elicited an anti-inflammatory response rather than a pro-inflammatory one. These results were consistent with those reported in the literature. The previous literature reported that the EcN-derived OMVs obtained via ultracentrifugation could induce stronger anti-inflammatory responses than pro-inflammatory responses in the RAW264.7 macrophages.

## 2. Materials and Methods

### 2.1. Materials and Apparatus

The RAW264.7 cells were purchased from the Shanghai Cell Biology Institute of the Chinese Academy of Sciences. Fetal bovine serum (FBS) and RPMI-1640 cell culture medium were purchased from Thermo Fisher Scientific (Waltham, MA, USA). RIPA buffer was purchased from Beyotime Biotechnology (Shanghai, China). The 0.3 μm filter membrane (PHWP02500) was purchased from Millipore Corp. (Darmstadt, Germany). Swinnex filter holder (SX0002500) was purchased from Millipore Corp. (Darmstadt, Germany). The 0.45 μm filter (PES membrane, SLHPR33RS) and ultrafiltration centrifuge tube (regenerated cellulose membrane, 100 kDa MWCO, UFC9100) were purchased from Millipore Corp. (Darmstadt, Germany).

The DiO membrane (C1038) and Hoechst 33,342 cellular nucleus dyes were purchased from Beyotime Biotechnology in China (Shanghai, China). The ELISA kits for TNF-α, IL-6, IL-1β, and IL-10 were purchased from Servicebio in China (Wuhan, China). The BCA protein assay kit was purchased from Beyotime Biotechnology in China (Shanghai, China). The SDS-PAGE gel preparation kit and loading buffer (5×) were purchased from Beyotime Biotechnology in China (Shanghai, China). Prestained protein molecular weight marker (26616) was purchased from Thermo Fisher Scientific (Thermo Scientific, Waltham, MA, USA). The G-250 protein stain reagent (WE0290) was purchased from Beijing Baiolaibo Technology Co., Ltd., located in China (Beijing, China). The LB growth medium contained 10 g peptone, 5 g yeast extract, 10 g NaCl, and 1 L of distilled water per liter. It was sterilized under high pressure at 121 °C for 20 min. The PBS buffer contained 10 mM Na_2_HPO_4_, 1.8 mM KH_2_PO_4_, 2.7 mM KCl, and 137 mM NaCl adjusted to pH 7.4. All PBS solutions used in this study were passed through a 0.22 μm membrane filter. The *Escherichia coli* Nissle 1917 (EcN) was purchased from Baosai Biological Company in China (Hangzhou, China).

The CO_2_ incubator was purchased from Thermo Fisher Scientific (Thermo Scientific, Waltham, MA, USA). All TEM images were acquired on a JEM1011 electron microscope at 80 kV (JEOL, Tokyo, Japan). NTA was performed on a Nano Sight NS300 instrument (Malvern, Worchestershire, UK). The SpectraMax M_5_ full-wavelength microplate reader was purchased from Molecular Devices (Molecular Devices, Sunnyvale, CA, USA). The Olympus lx83 inverted fluorescence microscope was purchased from Olympus (Olympus, Tokyo, Japan), and the ultra-high-speed refrigerated centrifuge (Optima XPN-100) was purchased from Beckman Coulter (Beckman, Los Angeles, CA, USA).

### 2.2. Measuring Growth Curve for EcN

The EcN glycerol cryopreservation solution (50 μL) was inoculated into 5 mL of sterile LB culture medium at a 1:100 ratio. This was cultured in a temperature-controlled shaking incubator at 37 °C and 200 rpm until the OD_600_ value of the bacterial solution was ~0.6. Two bottles of 100 mL sterile LB culture medium were prepared, and the seed bacterial liquid was inoculated into 100 mL of the sterile LB culture medium at a ratio of 1:100. One of the bottles was not inoculated and served as the negative control group. The bacteria were cultured at 37 °C and 200 rpm. The aliquots of 1 mL of the bacterial culture were removed at 0, 1.5, 3, 4, 6, 8, 10, 12, 14, 16, 18, 20, 22, and 24 h. Of these, 200 μL of each was added to different wells of a 96-well plate, with three duplicate wells for each time point. The OD_600_ value of each well was detected using the microplate reader to prepare the EcN growth curve.

### 2.3. EcN Culture and Isolation of EcN-Derived OMVs

The aliquots of EcN glycerol cryopreservation solution (100 μL) were inoculated into 10 mL of sterile LB culture medium at a 1:100 ratio before being cultured at 37 °C and 200 rpm for 8 h. Of this seed liquid, 250 μL was then inoculated into 250 mL of sterile LB culture medium. Two bottles were inoculated in this manner and cultured at 37 °C and 200 rpm for 18 h.

#### 2.3.1. EcN-Derived OMV Isolation via Gradient Filtration Method

The EcN culture medium was collected (500 mL). This was centrifuged at 15,000× *g* at 4 °C to remove the bacterial cells and sediments in the culture medium. The supernatant was filtered through a 0.45 µm filter to remove residual bacteria and large cellular debris, and then it was filtered through a 0.3 μm filter membrane. The collected filtrate was centrifuged through an ultrafiltration centrifuge tube (100 kDa) at 5000× *g* until the remaining volume was approximately 0.5 mL. Any residual protein was removed through three consecutive washes with PBS solution at 5000× *g* for 30 min each, with 12 mL of the PBS solution added during each wash. The final precipitates (i.e., purified EcN-derived OMVs) were dissolved in 500 μL of PBS and stored at −80 °C for later use.

#### 2.3.2. EcN-Derived OMV Isolation via Ultracentrifugation

The EcN culture medium was collected and centrifuged at 15,000× *g* for 20 min at 4 °C to remove the bacterial cells and sediments in the culture medium (500 mL). The supernatant was filtered through a 0.45 µm filter to remove residual bacteria and large cellular debris.

The filtrate was concentrated through an ultrafiltration centrifuge tube (100 kDa), and approximately 20 mL of the concentrated liquid was finally retained. The concentrated liquid was ultracentrifuged at 100,000× *g* for 1.5 h at 4 °C. The pellet was pipetted and washed with PBS (20 mL), followed by another centrifugation step at 100,000× *g* for 1.5 h at 4 °C. This supernatant was discarded, and the purified EcN-derived OMVs were resuspended in 500 μL of PBS and stored at −80 °C.

### 2.4. TEM of EcN-Derived OMV Samples

The EcN-derived OMV samples were loaded onto TEM copper grids and dried for 20 min. They were negatively stained using 2% uranyl acetate for 10 min before TEM images were acquired. TEM imaging was performed at 80 kV.

### 2.5. NTA of EcN-Derived OMV Samples

The EcN-derived OMVs were analyzed via NTA to determine their size distribution and concentration. They were warmed to room temperature and vortexed to break apart any aggregates. A 10 µL volume of EcN-derived OMVs sample was taken out and subsequently diluted with PBS buffer to achieve a concentration range from 1 × 10^7^ particles/mL to 1 × 10^9^ particles/mL. The size distribution and concentration of the OMVs were then determined using the particle matrix ZetaView PMX 110 with a 405 nm emission light.

### 2.6. Sodium Dodecyl Sulfate Poly-Acrylamide Gel Electrophoresis (SDS-PAGE)

Protein (20 μg) from the EcN-derived OMVs was boiled in SDS-PAGE loading buffer (5×) for 10 min. The samples were subsequently centrifuged (12,000× *g*), and the resulting supernatants were analyzed via SDS-PAGE (12% separating gel). The gel was stained using the G-250 protein stain reagent.

### 2.7. BCA Protein Quantification of EcN-Derived OMVs

The aliquots of 0, 1, 2, 4, 8, 12, 16, and 20 μL of a standard BSA solution (0.5 μg/μL) were diluted to concentrations of 0, 0.025, 0.05, 0.1, 0.2, 0.3, 0.4, and 0.5 μg/μL. If the resultant solutions were <20 μL, PBS buffer was added to make up the volume. A 200 μL aliquot of BCA solution was then added and incubated for 30 min at 37 °C, and the absorbance value at 562 nm was measured to build the standard curve. A certain amount of the EcN-derived OMV sample was mixed with RIPA lysis solution. The mixture was oscillated at room temperature for 15 min. A 2 µL volume of each EcN-derived OMV sample’s lysate was obtained to measure the absorbance at 562 nm according to the procedure described above. The concentration of the EcN-derived OMV sample was then determined according to the BCA standard curve and sample dilution factor.

### 2.8. EcN-Derived OMV Internalization by RAW264.7 Macrophages

A 10 μL aliquot of the EcN-derived OMV sample was diluted in 500 μL of PBS buffer and mixed with 0.5 μL of DiO membrane dye. For the negative control group, we used 500 μL of PBS buffer mixed with 0.5 μL of DiO membrane dye. The reaction solution was purified via an ultrafiltration centrifuge tube (100 kDa) at 6000 rpm for 30 min to remove excess dye. The precipitate was resuspended in 200 μL of PBS. The DiO-labeled EcN-derived OMVs were co-cultured with the RAW264.7 macrophages. After 16 h, the RAW264.7 cells were washed with the PBS buffer. They were then stained with Hoechst 33,342 for 5 min, washed with the PBS buffer, and added to the cell culture medium for fluorescence imaging.

### 2.9. Effect of the EcN-Derived OMVs on Cytokine Secretion in RAW264.7 Macrophages

The RAW264.7 cells were seeded into a 24-well plate at concentrations of 1 × 10^5^/well. A 1 mL aliquot of culture medium was added to each well, and the plate was cultured at 37 °C and 5% CO_2_ until the cell confluence was 70–80%. A 0.95 mL aliquot of culture medium was then added to each well to replace the culture medium, and 50 μL of the EcN-derived OMV sample at concentrations of 20, 40, 80, 160, and 320 μg/mL was added so that the final OMV concentrations in each well were 1, 2, 4, 8, and 16 μg/mL, respectively. After the RAW264.7 cells were co-cultured with these varying concentrations of EcN-derived OMVs for 16 h, the culture medium was collected and centrifuged at 1500× *g* for 10 min at 4 °C, and the supernatant was collected. The ELISA kit was used to detect the concentrations of pro-inflammatory cytokines (i.e., TNF-α, IL-6, and IL-1β) and anti-inflammatory cytokines (i.e., IL-10) in the supernatant, according to the manufacturer’s instructions.

### 2.10. Statistical Analysis

All statistical analyses were performed using GraphPad Prism 5.0 (GraphPad Software). Statistical analyses were performed using Student’s *t*-test and one-way ANOVA analysis of variance. Data are represented as the means ± standard deviations (SDs) of three independent experiments. * *p* < 0.05, ** *p* < 0.01, and *** *p* < 0.001.

## 3. Results

### 3.1. EcN-Derived OMV Isolation via Traditional Ultracentrifugation and Gradient Filtration Method

An ultrafiltration method that isolates exosomes from cell culture mediums has been reported. As described in the literature, the 100 kDa ultrafiltration membrane was used for the separation of exosomes [24]. Based on this precedent, we developed a gradient filtration method to purify OMVs from a Gram-negative bacterial culture medium. A schematic diagram of the gradient filtration is shown in Figure S1a. This method mainly relies on a combination of filter membranes of different sizes (300 nm and 100 kDa). Larger particles are trapped while small molecular substances pass through, thereby achieving a separatory effect. The Gram-negative bacterial culture mediums were filtered through membranes of varying sizes (300 nm and 100 kDa) to concentrate and purify the intact OMVs within the size range of 20−250 nm. This process effectively removed free proteins and other small molecules, and the concentrated OMVs were collected. The gradient filtration method has advantages such as simple operation, low cost, ability to handle large volumes of culture medium, and maintaining the biological activity of the OMVs post-separation. Currently, ultracentrifugation is the predominant method used for the isolation of OMVs. The gradient filtration method was evaluated in comparison to traditional ultracentrifugation in terms of the morphology and size distribution of the EcN-derived OMVs. We used the two different aforementioned methods to isolate the EcN-derived OMVs. The morphology of the EcN-derived OMVs was analyzed by means of transmission electron microscopy (TEM), and the size distribution of EcN-derived OMVs was analyzed by means of nanoparticle tracking analysis (NTA). The protein analysis of the EcN-derived OMVs obtained using the two different methods was carried out with SDS-PAGE.

**Figure 1:**
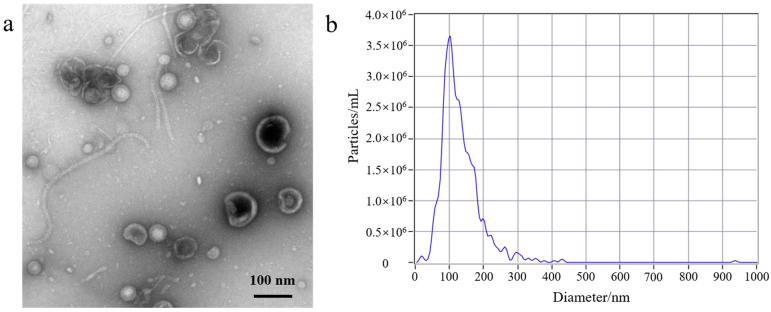

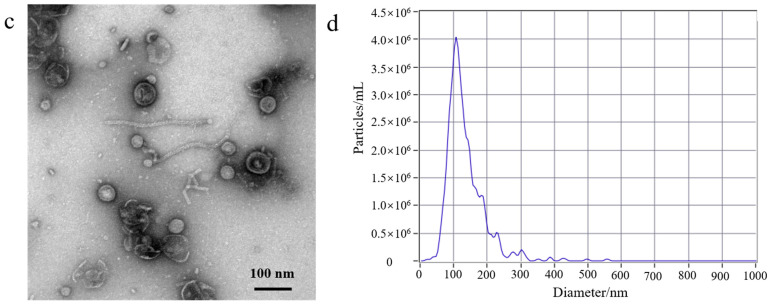
Transmission electron microscopy (TEM) and nanoparticle tracking analysis (NTA) of EcN-derived OMVs obtained through ultracentrifugation and the gradient filtration method. (**a**,**b**) Ultracentrifugation; (**c**,**d**) the gradient filtration method.

First, to better understand the growth characteristics of EcN, we measured the growth curve of EcN. The experiment results are shown in Figure S1b; 0–3 h was the lag phase for EcN, 4–14 h was its logarithmic growth phase, 16–22 h was the stable growth phase for EcN, and >22 h represented the death phase. EcN was cultured for a duration of 18 h, and the EcN-derived OMVs were isolated. Second, a volume of 500 mL of EcN culture medium was utilized for the purification experiment. The EcN-derived OMVs were isolated through traditional ultracentrifugation and through the gradient filtration method, respectively. Lastly, the morphological characteristics and particle sizes of the isolated EcN-derived OMVs were analyzed using TEM and NTA. As shown in Figure 1, The morphology of the EcN-derived OMVs obtained via the two different methods was clearly observed using TEM. The TEM results revealed that membrane structures with an uneven size distribution were detected in the isolated EcN-derived OMVs. The size distribution of EcN-derived OMVs obtained through the two different methods was basically consistent with that determined by means of NTA. The NTA results showed that the largest particle size of the collected EcN-derived OMVs was approximately 100 nm, with a size distribution ranging from 20 to 250 nm. Importantly, to further confirm the efficacy of the collected EcN-derived OMVs, the protein analysis of the EcN-derived OMVs obtained using the two methods was carried out with SDS-PAGE. The experimental results obtained using SDS-PAGE are shown in Figure S2. The results revealed that the abundance of proteins in the EcN-derived OMVs was unambiguously detected using SDS-PAGE for the two different methods. Notably, all of the protein bands identified in the EcN-derived OMVs obtained using the gradient filtration method corresponded to those observed via ultracentrifugation. These results were consistent with the particle sizes and structural characteristics of OMVs derived from other Gram-negative bacteria that have been reported in the literature and demonstrated that our gradient filtration method could efficiently isolate OMVs from culture medium containing Gram-negative bacteria [25,26].

### 3.2. Comparison of the Yield of EcN-Derived OMVs Isolated via Ultracentrifugation and the Gradient Filtration Method

**Figure 2:**
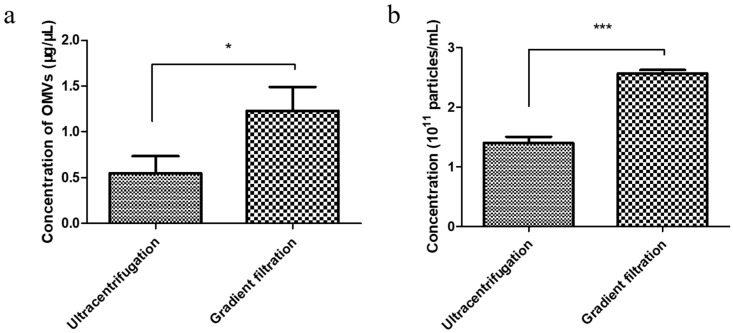
Comparison of the yields of the EcN-derived OMVs obtained using ultracentrifugation and the gradient filtration method through bicinchoninic acid (BCA) protein and NTA quantification. (**a**) BCA protein quantification. (**b**) NTA quantification. Data are represented as means ± SDs of three independent experiments. * *p* < 0.05, and *** *p* < 0.001, as determined using a Student’s *t*-test.

To determine the gradient filtration method’s efficiency, we performed a comparative yield experiment wherein the EcN-derived OMVs were isolated via both traditional ultracentrifugation and the gradient filtration method. The concentrations of the EcN-derived OMVs obtained using the two methods were compared through bicinchoninic acid (BCA) protein and NTA quantification, respectively. A volume of 250 mL of the EcN culture medium was used as the starting material for the purification experiment. First, BCA protein quantification of the EcN-derived OMVs obtained using the two methods was performed. The standard curve used for the BCA protein quantification method was measured as shown in Figure S3. As shown in Figure 2a, the concentration of EcN-derived OMVs obtained via ultracentrifugation was 0.55 ± 0.19 μg/μL, and the concentration obtained using the gradient filtration approach was 1.23 ± 0.26 μg/μL. Then, the yields of EcN-derived OMVs purified using the two different methods were compared through NTA quantification. As shown in Figure 2b and Figure S4, the yields of the EcN-derived OMVs obtained via traditional ultracentrifugation and the gradient filtration method were 1.40 ± 0.10 × 10^11^ particles/mL and 2.60 ± 0.06 × 10^11^ particles/mL, respectively. Therefore, the gradient filtration method was about twice as efficient as the traditional ultracentrifugation. These results indicated that the gradient filtration method is a more cost-effective, efficient, and convenient approach for isolating OMVs from Gram-negative bacterial culture medium compared to traditional ultracentrifugation, demonstrating its potential for industrial-scale production.

### 3.3. EcN-Derived OMVs Obtained via the Gradient Filtration Method Were Taken up by RAW264.7 Macrophages

**Figure 3:**
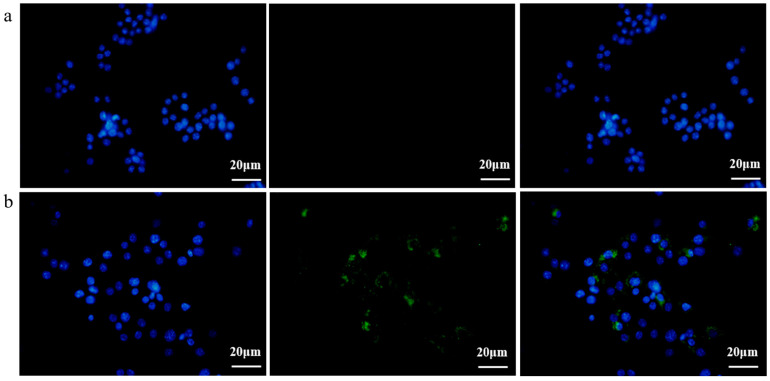
Fluorescence imaging of the RAW264.7 macrophages incubated with the DiO-labeled EcN-derived OMVs obtained via the gradient filtration method after 16 h (scale bars = 20 µm). (**a**) Negative control group without DiO-labeled EcN-derived OMVs; (**b**) experimental group containing DiO-labeled EcN-derived OMVs. DiO (green) was used for cell membrane staining. Hoechst 33,342 (blue) was used for nuclear staining. Representative images from three independent experiments are presented.

Subsequently, in order to investigate the EcN-derived OMVs acquired via the gradient filtration method without inhibiting their biological activity, a fluorescence imaging experiment was conducted to observe the internalization of these EcN-derived OMVs by the RAW264.7 macrophages. EcN-derived OMVs acquired via the gradient filtration method were labeled with 3,3′-dioctadecyloxacarbocyanine perchlorate (DiO) cell membrane dye and incubated with the RAW264.7 macrophages for 16 h. Phosphate-buffered saline (PBS) was mixed with DiO and purified through ultrafiltration to serve as the negative control group. As shown in Figure 3a,b, the RAW264.7 macrophages in the negative control group were free of the DiO-labeled EcN-derived OMVs, so no fluorescence was observed in them after 16 h of incubation. By contrast, the RAW264.7 macrophages in the experimental group took up the DiO-labeled EcN-derived OMVs and thus showed green fluorescent intracellular spots after 16 h of incubation. These results demonstrated that the EcN-derived OMVs acquired via the gradient filtration method could be internalized by the RAW264.7 macrophages to transmit relevant molecular signals. Thus, the internalization experiments provided additional evidence that the gradient filtration method would not inhibit the biological activity of the EcN-derived OMVs.

### 3.4. EcN-Derived OMVs Obtained via the Gradient Filtration Method Induced Secretion of Inflammatory Cytokines by RAW264.7 Macrophages

**Figure 4:**
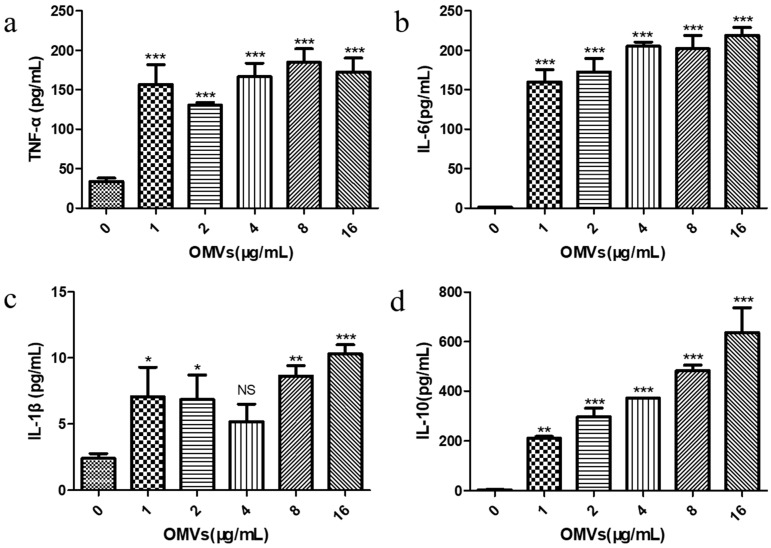
Effect of stimulation with the EcN-derived OMVs obtained via the gradient filtration method (0, 1, 2, 4, 8, and 16 μg/mL) on the secretion of pro-inflammatory and anti-inflammatory cytokines by RAW264.7 macrophages; (**a**) TNF-α; (**b**) IL-6; (**c**) IL-1β; (**d**) IL-10. Data are represented as means ± SDs of three independent experiments. * *p* < 0.05, ** *p* < 0.01, and *** *p* < 0.001 were determined via one-way analysis of variance (ANOVA). All the statistics were performed between the 0 μg/mL of EcN-derived OMV group and the others.

Previous studies have reported that the inflammatory response is the result of the body’s innate immunity, which is mainly triggered by the activation of pro-inflammatory signaling cascades from macrophages [27]. Cytokines produced by macrophages can cause this cascade reaction, thereby regulating the host’s immune response. Cytokines involved in the inflammatory response include both pro-inflammatory cytokines (e.g., TNF-α, IL-6, and IL-1β) and anti-inflammatory cytokine (e.g., IL-10) [28]. In order to explore the immune response of the RAW264.7 macrophages stimulated by the EcN-derived OMVs obtained via the gradient filtration method, we examined the effects of stimulation at different concentrations (0, 1, 2, 4, 8, and 16 μg/mL), in terms of the secretion of both pro-inflammatory cytokines (TNF-α, IL-6, and IL-1β) and anti-inflammatory cytokine (IL-10) by the RAW264.7 macrophages, via enzyme-linked immunosorbent assay (ELISA). The standard curves for the ELISA experiment are shown in Figure S5. As shown in Figure 4a–c, compared with the PBS control group (0 μg/mL of EcN-derived OMVs), the levels of TNF-α, IL-6, and IL-1β produced by the RAW264.7 macrophages treated with the EcN-derived OMVs obtained via the gradient filtration method were significantly higher at all concentrations tested. It was found that just 1 μg/mL of the EcN-derived OMVs could significantly increase the secretion of TNF-α (156.83 ± 24.86 pg/mL), IL-6 (160.13 ± 15.51 pg/mL), and IL-1β (7.07 ± 2.22 pg/mL). The TNF-α concentration was the highest in the 8 μg/mL group (184.60 ± 17.40 pg/mL), and the levels of IL-6 (219.11 ± 9.93 pg/mL) and IL-1β (10.31 ± 0.70 pg/mL) were the highest in the 16 μg/mL group. Notably, as shown in Figure 4d, the concentration of anti-inflammatory IL-10 produced by the EcN-derived OMV-treated RAW264.7 macrophages also increased significantly, with the 16 μg/mL group having the highest content (636.2 ± 100.80 pg/mL). Furthermore, the concentration of anti-inflammatory cytokines (IL-10) was higher than that of pro-inflammatory cytokines (TNF-α, IL-6, and IL-1β) in each OMV group. These results suggested that EcN-derived OMVs obtained via the gradient filtration method could induce RAW264.7 macrophages to secrete pro-inflammatory cytokines such as TNF-α, IL-6, and IL-1β, as well as the anti-inflammatory cytokine IL-10. Moreover, the EcN-derived OMVs induced more anti-inflammatory responses than pro-inflammatory responses. These results were consistent with previous results in the literature. The related literature reported that EcN-derived OMVs obtained via ultracentrifugation had the capacity to induce more anti-inflammatory responses than pro-inflammatory responses in RAW264.7 macrophages [29]. Therefore, the above experimental results further indicated that the gradient filtration method could efficiently isolate OMVs from the culture medium of Gram-negative bacteria.

## 4. Discussion

Studies have reported that almost all Gram-negative bacteria can secrete bacterial extracellular vesicles (bEVs) [30]. bEVs secreted by Gram-negative bacteria are referred to as outer membrane vesicles (OMVs) due to their origin (the outer membrane) [1,2]. OMVs are spherical vesicles with lipid membrane structures and particle sizes of ~20–250 nm [1]. OMVs can carry lipopolysaccharide (LPS), peptidoglycan, lipids, proteins, nucleic acids, toxins, virulence factors, and other substances derived from their parent bacterial cells. As carriers, OMVs can ensure the long-distance delivery of bacterial active ingredients without requiring direct contact between cells [31]. At present, the separation and purification of OMVs are carried out mainly through ultracentrifugation and density gradient centrifugation. However, those traditional methods are inefficient, inconvenient, and costly. Liu et al. have reported a size-based exosome isolation tool that is characterized by its simplicity, ease of use, and ability to efficiently isolate exosomes with high yield and purity from various biofluids, including culture media, plasma, and urine [32]. We developed a new method for purifying OMVs based on gradient filtration. The method was applied to isolate OMVs from a liquid culture of EcN bacteria. EcN is one of the few Gram-negative bacteria used as a probiotic [33]. Related research indicates that EcN is mainly used to treat various gastrointestinal dysfunctions, including Crohn’s disease and ulcerative colitis [34]. On the one hand, EcN can colonize the human intestine and prevent pathogenic bacteria from invading the intestinal mucosa, thereby exerting a protective and repairing effect on the intestinal mucosal barrier [35]. On the other hand, EcN also participates in the body’s immune regulation, balances the secretion of immune factors, enhances host immunity, and thereby alleviates and treats inflammation [36,37,38]. First, we used traditional ultracentrifugation and the gradient filtration method to isolate EcN-derived OMVs. By measuring the growth curve of EcN, we determined that the optimal time to isolate OMVs was when EcN was cultured for a duration of 18 h. The morphology of the EcN-derived OMVs isolated through the two different methods was observed via TEM. The size distribution of EcN-derived OMVs obtained via the two different methods was measured via NTA. The TEM results showed that our purified EcN-derived OMVs were ~100 nm in size and had lipid membrane structures. Their morphological characteristics and particle sizes were further confirmed through NTA and found to be consistent with those reported in the literature [25,26]. Second, we compared the yields of the purified EcN-derived OMVs through traditional ultracentrifugation vs. our novel gradient filtration method. BCA protein quantification results showed that the concentration of the EcN-derived OMVs obtained via ultracentrifugation was 0.55 ± 0.19 μg/μL, and the concentration obtained using the gradient filtration approach was 1.23 ± 0.26 μg/μL. Simultaneously, the NTA quantification results showed that the yields of the EcN-derived OMVs obtained using traditional ultracentrifugation and the gradient filtration method were 1.40 ± 0.10 × 10^11^ particles/mL and 2.60 ± 0.06 × 10^11^ particles/mL. The above results confirmed that the efficiency of the gradient filtration approach was about twice that of traditional ultracentrifugation for this application. Finally, the EcN-derived OMVs obtained through gradient filtration were incubated with RAW264.7 macrophages for fluorescence imaging of internalization experiments. We observed that the EcN-derived OMVs could be internalized by the RAW264.7 macrophages, which indicated that the biological activity of EcN-derived OMVs was not inhibited. Those results were consistent with previous results in the literature. The related literature has reported that OMVs derived from *Legionella pneumophila* can interact with RAW264.7 macrophages in the body and that OMVs are present in the cytoplasms of macrophages in the lung tissue of patients infected with *Legionella pneumophila* [39]. Compared with conventional methods that can be cumbersome, time-consuming, and costly, our approach is fast, convenient, suitable for achieving industrial-scale yields for mass production, and maintains the biological functional activity of the purified OMVs.

Cytokines are proteins or peptides with immunomodulatory functions that can change the behaviors of themselves or other cells. They can serve as effector molecules of the body’s innate immune response [40,41]. The results of this study showed that the levels of cytokines secreted by RAW264.7 macrophages in our PBS control group (0 μg/mL of EcN-derived OMVs) were very low, with the pro-inflammatory cytokine IL-6 and anti-inflammatory cytokine IL-10 being essentially undetectable in the cellular supernatant. However, the RAW264.7 macrophage groups treated with different concentrations of our purified EcN-derived OMVs obtained via the gradient filtration method (i.e., 1, 2, 4, 8, and 16 μg/mL) showed significantly increased production of the pro-inflammatory cytokines TNF-α, IL-6, and IL-1β. Both TNF-α and IL-1β are early regulators of the inflammatory response that can induce the secondary release of other cytokines. Treatment with just 1 μg/mL of EcN-derived OMVs obtained via the gradient filtration method was shown to promote the large-scale secretion of TNF-α and IL-1β in RAW264.7 macrophages. These treated RAW264.7 macrophages also increased their secretion of the anti-inflammatory cytokine IL-10. IL-10 is a type II cytokine that mediates immunosuppression [42]. IL-10 secreted by macrophages can inhibit the excessive synthesis of pro-inflammatory factors and maintain the balance between pro-inflammatory and anti-inflammatory immune responses. Furthermore, our experimental results showed that the anti-inflammatory response (i.e., IL-10) was greater than the pro-inflammatory response (i.e., TNF-α, IL-6, and IL-1β) in each EcN-derived OMV group, meaning these vesicles were more conducive to enhancing the anti-inflammatory functions of RAW264.7 macrophages. Relevant studies have reported that the use of 1 μg/mL of EcN-derived OMVs obtained through ultracentrifugation could stimulate RAW264.7 macrophages to increase their levels of immune-related enzymatic and phagocytosis activity, as well as their anti-inflammatory responses relative to pro-inflammatory ones [29]. These findings are fully consistent with our experimental results.

## 5. Conclusions

We successfully developed a new method for separating OMVs based on gradient filtration. This method is fast, convenient, low-cost, and capable of achieving the industrial-scale yields required for mass production, and it maintains biological activity in isolated OMVs. We compared the gradient filtration method with traditional ultracentrifugation to purify OMVs from the EcN bacterial culture medium and found that the novel method was about twice as efficient as the current standard ultracentrifugation method for this application. We then used RAW264.7 macrophages as an in vitro model to study the effects of the EcN-derived OMVs obtained through the gradient filtration method on the immunoregulatory functions of the RAW264.7 macrophages. We found that the purified EcN-derived OMVs were successfully internalized by the RAW264.7 macrophages and induced them to secrete the pro-inflammatory cytokines TNF-α, IL-6, and IL-1β, as well as the anti-inflammatory cytokine IL-10. We also found that the EcN-derived OMVs induced more anti-inflammatory response (i.e., IL-10) than pro-inflammatory response (i.e., TNF-α, IL-6, and IL-1β) in the RAW264.7 macrophages. These results were consistent with previous results in the literature. The related literature reported that EcN-derived OMVs obtained through ultracentrifugation could induce stronger anti-inflammatory responses than pro-inflammatory responses in RAW264.7 macrophages. Therefore, our new method for isolating OMVs has potentially broad applications in future OMV research.

## Data Availability

All data used for this study are contained within its text and Supplementary Materials.

## Supplementary Materials

The following supporting information can be downloaded at: https://www.mdpi.com/article/10.3390/membranes14060135/s1. Figure S1, Isolation of *Escherichia coli* Nissle 1917 (EcN)-derived bacterial outer membrane vesicles (OMVs). Figure S2, Protein analysis of the EcN-derived OMVs obtained using the two different methods was carried out with SDS-PAGE. Figure S3, Standard curve of bicinchoninic acid (BCA) protein quantification. Figure S4, Concentration of EcN-derived OMVs obtained using ultracentrifugation and the gradient filtration method through nanoparticle tracking analysis (NTA) quantification. Figure S5, Standard curve of determining pro-inflammatory and anti-inflammatory cytokine concentrations through ELISA.

## Abbreviations

bEVs	bacterial extracellular vesicles
OMVs	outer membrane vesicles
EcN	*Escherichia coli* Nissle 1917
TEM	transmission electron microscope
NTA	nanoparticle tracking analysis
SDS-PAGE	sodium dodecyl sulfate poly-acrylamide gel electrophoresis
BCA	bicinchoninic acid
DiO	3,3′-dioctadecyloxacarbocyanine perchlorate
TNF-α	tumor necrosis factor-alpha
IL-6	interleukin-6
IL-1β	interleukin-1β
IL-10	interleukin-10
LPS	lipopolysaccharide
NO	nitric oxide
ELISA	enzyme-linked immunosorbent assay
SDs	standard deviations
