# Supplementary material for: Efficient Isolation of Outer Membrane Vesicles (OMVs) Secreted by Gram-Negative Bacteria via a Novel Gradient Filtration Method
Source: Membranes (Basel). 2024 Jun 6;14(6):135. doi: 10.3390/membranes14060135 (PMC11205348; doi:10.3390/membranes14060135)
Supplement: Supplementary file 1 [file membranes-14-00135-s001.zip › membranes-2986238-supplementary.pdf]

Supplementary Materials

Efficient isolation of outer membrane vesicles (OMVs) secreted by Gram-negative bacteria via a novel gradient filtration method

Ning Li*, Minghui Wu, Lu Wang, Mengyu Tang, Hongbo Xin and Keyu Deng*

The national Engineering Research Center for Bioengineering Drugs and the Technologies, Institute of Translational Medicine, Nanchang University, Nanchang, 330031, China; 15179421660@163.com (M.W.); 407400210001@email.ncu.edu.cn (L.W.); 405600210073@email.ncu.edu.cn (M.T.); xinhb@ncu.edu.cn (H.X.)
* Correspondence: lining@ncu.edu.cn (N.L.); dky@ncu.edu.cn (K.D.)

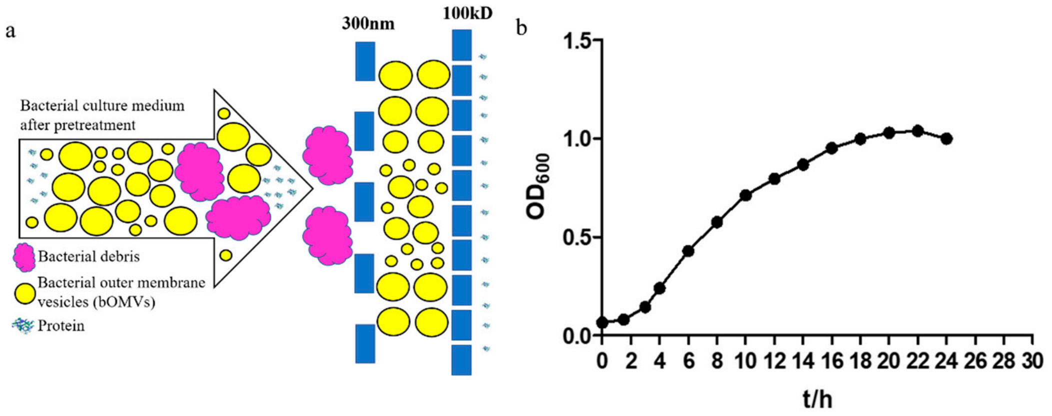

Figure S1. Isolation of *Escherichia coli* Nissle 1917 (EcN)-derived bacterial outer membrane vesicles (OMVs). (a) Schematic diagram of the gradient filtration method; (b) Growth curve of EcN.

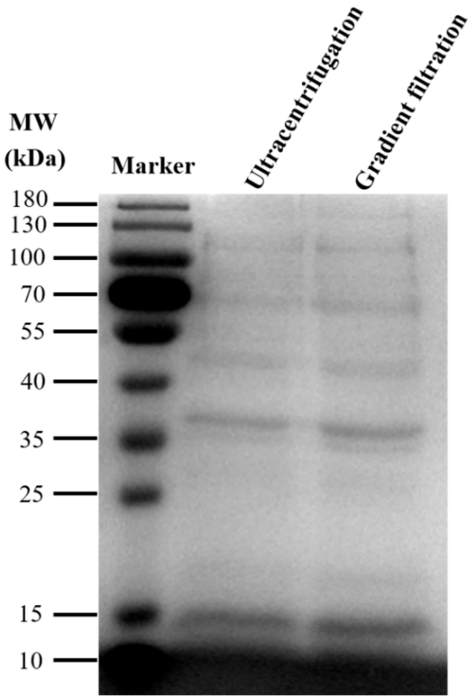

Figure S2. Protein analysis of the EcN-derived OMVs obtained using the two different methods was carried out with SDS-PAGE.

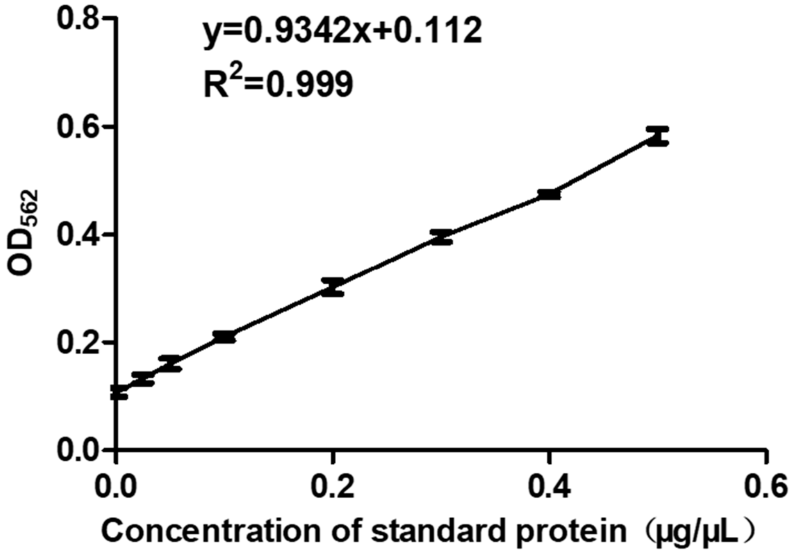

Figure S3. Standard curve of bicinchoninic acid (BCA) protein quantification.

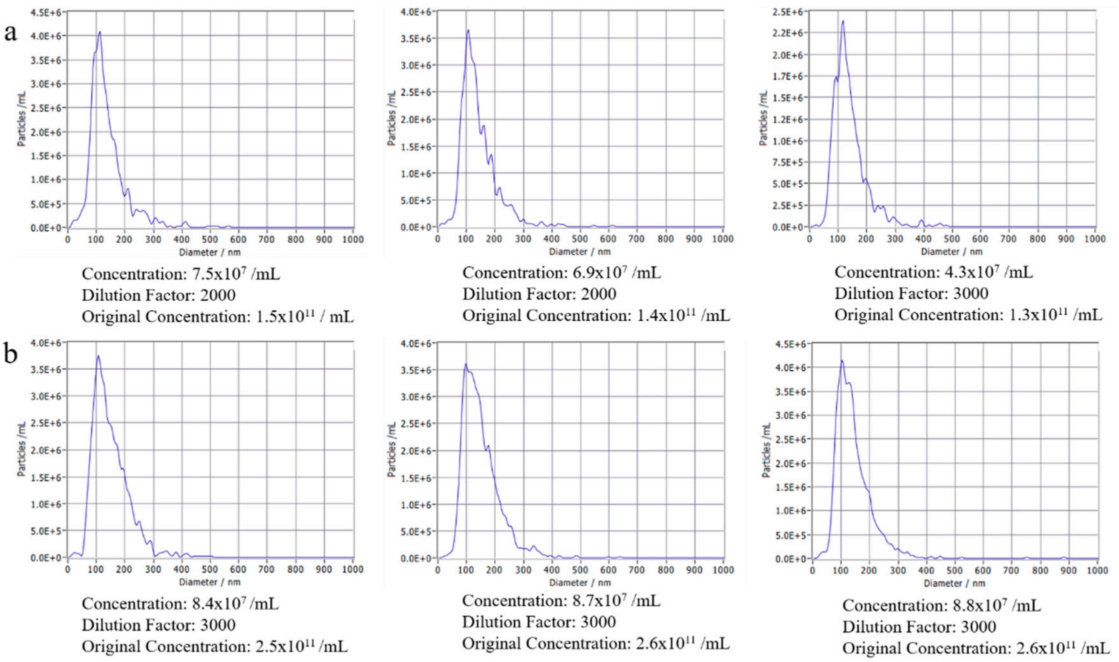

Figure S4. Concentration of EcN-derived OMVs obtained using ultracentrifugation and the gradient filtration method through nanoparticle tracking analysis (NTA) quantification. (a) ultracentrifugation; (b) Gradient filtration method. Data are representative of three independent experiments.

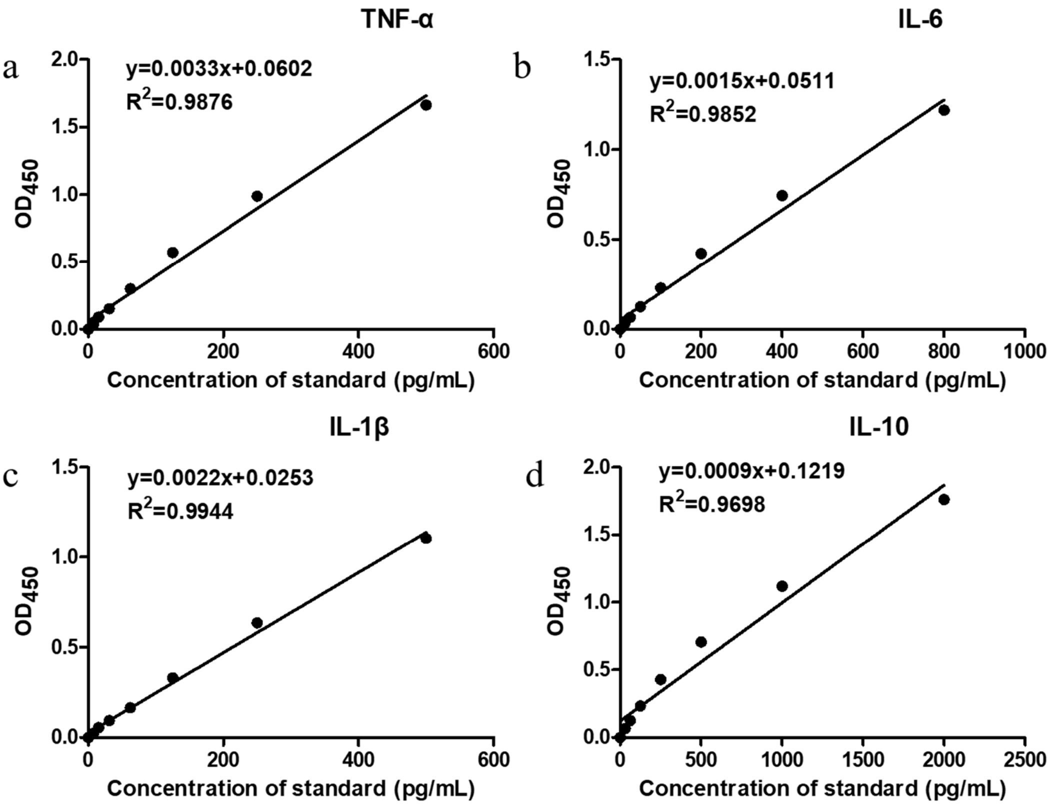

Figure S5. Standard curve of determining pro-inflammatory and anti-inflammatory cytokine concentrations through ELISA. (a) Standard curve of TNF-α; (b) Standard curve of IL-6; (c) Standard curve of IL-1β; (d) Standard curve of IL-10.
